# The moth *Hylesia metabus* and French Guiana lepidopterism: centenary of a public health concern

**DOI:** 10.1051/parasite/2012192117

**Published:** 2012-05-15

**Authors:** F. Jourdain, R. Girod, J.M. Vassal, F. Chandre, C. Lagneau, F. Fouque, D. Guiral, J. Raude, V. Robert

**Affiliations:** 1 Centre National d’Expertise sur les Vecteurs BP 64501 34394 Montpellier Cedex 5 France; 2 Institut Pasteur de Guyane BP 6010 97306 Cayenne France; 3 CIRAD, Campus international de Baillarguet 34398 Montpellier Cedex France; 4 Institut de Recherche pour le Développement, MIVEGEC, UMR IRD 224 – CNRS 5290 – UM1 – UM2 BP 64501 34394 Montpellier Cedex 5 France; 5 EID Méditerranée 165, avenue Paul Rimbaud 34184 Montpellier Cedex 4 France; 6 Institut Pasteur de la Guadeloupe, Laboratoire d’Entomologie médicale BP 484, Morne Jolivière 97183 Les Abymes Cedex France; 7 IMEP UMR Université P. Cézanne CNRS IRD, Faculté des sciences de Saint-Jérôme Boîte 441 13397 Marseille Cedex 20 France; 8 EHESP Avenue du Pr Léon Bernard 35043 Rennes France

**Keywords:** review, *Hylesia metabus*, French Guiana, lepidopterism, yellowtail moth dermatitis, nuisance, Lepidoptera, mangrove swamp, infestation, dermite, urticariad, revue, *Hylesia metabus*, papillon cendre, Guyane française, papillonite, nuisance, Lepidoptera, mangrove, pullulation, dermatite, urticaire

## Abstract

The females of the moths *Hylesia metabus* have their abdomens covered by urticating hairs looking like micro-arrows and causing a puriginous dermatitis to humans known as “papillonite” in French Guiana and also called yellowtail moth dermatitis or Caripito itch. The densities of the moths show great seasonal and annual variations depending on mechanisms mostly unknown. When *H. metabus* infestations occur, numerous cases of dermatologic manifestations are reported from people living near the mangrove swamps where the moths are developing. One hundred years after the first “papillonite” epidemic reported from French Guiana in 1912, the data presented herein summarize the actual state of knowledge on *H. metabus* biology and ecology and on the lepidopterism. Some recommendations are proposed for the surveillance and warning systems of *H. metabus* infestations and to avoid contact with the moths. Research priorities are suggested to improve the control against this problem emerging between nuisance and public health.

## Abstract (fr)

L’abdomen des femelles du papillon de nuit *Hylesia metabus* est recouvert de micro-fléchettes venimeuses qui déclenchent chez l’homme une dermatite urticarienne prurigineuse, dénommée “papillonite” en Guyane. Les densités de population de cette espèce sont extrêmement variables, sous la dépendance de mécanismes qui restent mal connus. Lors des pullulations, de nombreux cas de dermatites sont observés au sein des populations humaines vivant à proximité de la mangrove où se développent les chenilles et d’où émergent les papillons. Cet article dresse un état des connaissances, un siècle après la première épidémie de papillonite connue, observée en Guyane en 1912. La biologie et l’écologie du papillon sont présentées, ainsi que les aspects dermatologiques. La surveillance et l’alerte concernant les pullulations font l’objet de recommandations. Enfin, des pistes de recherche sont avancées pour améliorer les stratégies et méthodes de lutte utilisables contre ce papillon.

## Introduction

The first epidemic of Caripito itch was reported from French Guiana in 1912 (Boyé, 1932), based on observations of the physicians Devez and Henry describing the simultaneous occurrence of dermatitis human cases and “bad moths” infestations. The first clinical description of the urticarial dermatitis caused by *Hylesia metabus* (Cramer, 1775) was also made in French Guiana (Léger & Mouzels, 1918). One hundred years after these first reports, the epidemics of dermatitis caused by *H. metabus* are still recurrent in French Guiana. In 2011, an *H. metabus* infestation started in June in the small towns of Sinnamary and Iracoubo (French Guiana) and created a strong nuisance with dermatitis problem to their inhabitants and visitors. This episode was estimated exceptional for its duration and intensity and was followed by a mobilization of the population requesting the immediate implementation of control measures. Consequently, the “Direction Générale de la Santé” at the French Ministry of Health commissioned an advice to the newly created “Centre National d’Expertise sur les Vecteurs”. This advice (CNEV, 2011) was based on the knowledge acquired on *H. metabus* during this expertise by a working group, with special emphasis on the surveillance and control tools. This advice pointed out the lack of scientific data on *H. metabus* bionomics and on the infestation driving variables. Both should be deeply investigated to better control this problem.

## Lepidopterism

Most of the Lepidoptera species, either as caterpillars or adults are considered of little medical importance because they are not vectors of pathogens for humans and animals and not responsible of nuisance. Consequently this group is the matter of few studies in the area of medical or veterinary entomology. However, among this large group of insects, a few of them can cause severe clinical manifestations, mostly dermatologic injuries, known as lepidopterism. The venomous Lepidoptera are classified into two groups, the phanerotoxic and the cryptotoxic species according to the presence or absence respectively of an external apparatus (hairs, setae) producing the toxic substance (Vassal, 1989; Diaz, 2005). The cryptotoxic species are harmful only through ingestion. In France, most of the dermatologic manifestations due to Lepidoptera are caused by caterpillars such as the processionary pine caterpillars (*Thaumetopoea pityocampa*). However, contact with adults can also result in urticant or vesicant clinical manifestations. For other species such as *H. metabus*, both caterpillars and adults are dangerous for humans. According to the stage of the insect, caterpillar or adult, the dermatitis is called erucism or lepidopterism respectively.

Cases of lepidopterism are reported worldwide, in Africa due to the moths *Anaphe* sp., in Asia with *Euproctis* sp., in Australia with the Zygaenidae family and also in Europe as described above (Lamy & Werno, 1989; Hossler, 2010; Battisti *et al.*, 2011). But, the most severe manifestations are observed in Southand Central-America, all caused by species of the genus *Hylesia* (Fornés & Hernández, 2000; Lemaire, 2002; Willat *et al.*, 2003; Iserhard *et al.*, 2007). In French Guiana, lepidopterism epidemics are caused by the species *H. metabus*, also called “papillon cendre” *i.e.* ashen moth or “yellowtail moth”, respectively due to the urticant micro-arrow setae (“ashes”) that are thrown away when flying and to the colour of the abdomen. This phanerotoxic Lepidoptera is responsible of the lepidopterism called “yellowtail moth dermatitis”. This affection is known as “papillonite” in French Guiana (Tisseuil, 1935) and “Caripito itch” in Venezuela from the port town of Caripito providing in Venezuela the first identification of numerous clinical manifestations (Dinehart *et al.*, 1985).

## Taxonomy and systematics of *H. metabus*

The genus *Hylesia* includes about 110 species with 28 species reported from French Guiana (Lemaire, 2002). However, this list is changing because two other species had been recently observed in French Guiana: *Hylesia moronensis* Lemaire 1976 and *Hylesia humilis* Dogin 1923 (F. Bénéluz, pers. com.). *Hylesia metabus* belongs to the Family Saturniidae (= Attacidae) and Sub-Family Hemileucinae. *Hylesia urticans* is synonymous of *Hylesia metabus*. This moth is commonly called “palometa peluda” in Venezuela. Sexual dimorphism is observed among the adults, with females being bigger with short stub-like antennae and a voluminous abdomen covered by urticating micro-arrow setae. Males are smaller with long dendritic antennae and no-urticating setae.

## Urticating apparatus of H*. metabus*

Tisseuil (1935) made the first identification of the females of *H. metabus* as the causing agents of the “papillonite” dermatitis. He observed that a skin contact with the *H. metabus* caterpillars was followed by an urticarial dermatologic manifestation, in red patch, localized at the contact point. The same contact with a male moth was followed by a similar reaction that persisted for a few hours. The skin reactions with the *H. metabus* caterpillars and males were in all cases less harmful compared with the dermatologic manifestations due to contacts with the females.

The urticant apparatus of the *H. metabus* female is constituted by micro-arrow setae or spines covering the abdomen (Lamy & Lemaire, 1983). The primary function of the urticant setae is to cover the batch of eggs and protect the nest from predators. These setae are produced by differentiated cells (called trichogene and tormogene cells), forming a monocell hypoderm similar to the hypoderm producing the scales. These setae can thus be assimilated to transformed scales (Novak *et al.*, 1987; Pelissou & Lamy, 1988) or true setae (Battisti *et al.*, 2011). Rodriguez *et al.* (2004) described four types of abdominal setae for the *H. metabus* female, among which two are urticant. The setae S1 and S2, 2,000 and 155 μm long respectively, and on dorsal position, are not urticant. The setae S1, S3 and S4 are found on ventral position. The S3 setae, 190 μm long and 8 μm wide are straight with thin barbed tips apex orientated and are urticant. These micro-arrow setae cover densely the abdominal segments (about 50,000 setae/mm2 on the abdominal segments 4 to 7) (Lamy *et al.*, 1982; Pelissou & Lamy, 1988; Rodriguez *et al.*, 2004). The S3 setae have an apical drain that may deliver the urticant substance, even without being broken (Lamy *et al.*, 1984). The S4 setae, 1 mm long and 60 μm wide, are arc-shaped, flat, with thin barbed tips apex orientated, and are also urticant. The apex is differentiated in a thin cylindrical tube delivering the urticant substance. The S4 setae are the only setae covering the sides of the female abdomen with a density of about 3,500 setae/mm2. The S4 setae are the protective setae for the egg batches. The base of the S3 and S4 setae is lightly attached to the abdominal tegument, so they can detach easily and be thrown away. The S1 setae are the only setae found on the abdomen of the *H. metabus* males.

The composition of the urticant substance has been investigated with contradictory results. Some of them proposed a role for the histamine (Dinehart *et al.*, 1987) and other studies did not (Benaim-Pinto *et al.*, 1992; Vassal, 1989; Lundberg *et al.*, 2007). However, the clinical manifestations such as the necrosis of epidermic and dermic cells and the delayed cutaneous manifestations, suggest the presence of proteolytic enzymes (Benaim-Pinto *et al.*, 2002; Battisti *et al.*, 2011). Lundberg *et al.* (2002, 2007) have partially isolated a serine protease with kallikrein-like action. This enzyme increases the vascular permeability and creates inflammation and pains at the inoculation point. But, according to Lundberg *et al.* (2007), other proteins are involved in the mechanisms of action of the venomous substance.

## Clinical manifestations of the *H. metabus* dermatitis

The “papillonite” dermatitis caused by *H. metabus* is not fatal and only rare cases are hospitalized. The “papillonite” is thus mostly considered as a nuisance and not as major public health problem. This attitude has important consequences in decisionmaking terms for prevention and control measures.

The skin contact with urticating micro-arrow setae causes several clinical symptoms and some of them are severe. A large range of dermatological manifestations has been reported (Benaim-Pinto, 2002). The first manifestations are mainly an urticaria or papulovesicular dermatitis, occurring about 15 to 20 minutes after the exposition, and persisting for about seven days (Thiéry *et al.*, 2008). In some observations, the dermatitis persists from 12 hours to one year (Paniz-Mondolfi *et al*., 2011). Ducombs *et al.* (1983) reported that the injuries disappear in one or two days for the French Guiana inhabitants and can persist for seven days in the newly arrived population, in particular after a first contact. A delayed inflammatory cutaneous reaction is possible with bacterial secondary infection consecutive to itching.

The contamination happens through a direct skin contact with the moths or an indirect contact with the micro-arrow setae transported by winds or laid down in the environment (on furniture, towels, clothes, dishes, spider web, swamps water, etc.). The microarrow setae can keep their urticant nature several weeks, and maybe years after being detached from the moth. Indeed, a man experienced a serious hypersensitivity reaction when using its air conditioning system two months after the moth infestation (Germanetto, 1983). Vassal (1989) also reports a cutaneous reaction after handling a female that was kept in collection since five years. Secondary injuries can also appear in cutaneous areas that were not exposed to the urticant setae, through manual transportation and/or toxin diffusion with sweat (Ducombs *et al.*, 1983). Eyes complications were also observed with weeping, photosensitivity, conjunctivitis and keratitis (Ducombs *et al.*, 1983). In a few case, respiratory tract affection was reported on susceptible persons or because the upper respiratory tract were directly exposed (Ducombs *et al.*, 1983; Dinehart *et al.*, 1985), but no long-term manifestations were observed (Rodriguez-Morales *et al.* 2005). Only one Quincke’s oedema has been reported (Michel, 1981 *in* Ducombs *et al.*, 1983) and even if no anaphylactic shock was mentioned, some authors do not exclude this event (Paniz-Mondolfi *et al.*, 2011). Other systemic manifestations can also occur, such as nausea, fever, dizziness, tiredness and arthralgia (Rodriguez-Morales *et al.*, 2005; Lundberg *et al.*, 2007; Paniz-Mondolfi *et al.*, 2011).

## Therapeutics

The medical treatment of the dermatitis manifestations due to the contact with *H. metabus* toxins is essentially symptomatic (Hassing & Bauer, 2008; Hossler, 2010; Paniz-Mondolfi *et al.*, 2011). The methods usually employed for the treatment of allergic reactions seem poorly efficient (Germanetto, 1983; Thiéry *et al.*, 2008; Paniz-Mondolfi *et al.*, 2011) and topic corticoids or oral use of anti-histaminic substances have a low efficacy (Hossler, 2010). Some physical actions can relieve the patient pains, such as a very hot bath immediately after the exposition, maybe because of the thermolabile nature of the venomous substance. Frictions with aqueous solution of sodium hyposulfite (40-50 %) or cold water applied just after the first symptoms appearance can also relieve the itch. Other natural solutions were also reported, such as limejuice against the cutaneous symptoms, but the real efficacy of these means was not scientifically evaluated (Thiéry *et al.*, 2008). Finally, an early extraction of the micro-arrow setae with adhesive rubber was also proven to relieve itching manifestations (Paniz-Mondolfi *et al.*, 2011).

## Ecology of *H. metabus*

The ecology of the moth *H. metabus* in French Guiana was studied by Boyé (1932) and Vassal (1989). Now, only scientific teams of Venezuela are carrying out research studies on this moth, through a multidisciplinary project (Fornés & Hernández, 2000; Osborn, 2005a; Sociedad Venezolana de Entomología, 2005; Sociedad venezolana de Entomología, 2007).

### Geographical distribution

The genus *Hylesia* is exclusively neotropical and extensively distributed from north of Mexico to Argentina. According to Lemaire (2002), the species *H. metabus* is frequent at low altitudes in all South- America (French Guiana, Venezuela, Brazil, Ecuador, Peru and Bolivia) with the exception of the pacific side of the Andes Mountains, the southeast of Brazil and the semi-arid and arid zones. However, Vassal (1989) found *H. metabus* in very small densities in the Amazonian forest. All authors agree on the development of moth infestations only in the coastal areas covered by mangrove between the Orinoco (north) and Amazon (south) deltas (Germanetto, 1983; Hudson, 1985; Vassal, 1989; Fornés & Hernández, 2001) and also in Trinidad and Tobago (Polar *et al.*, 2010).

### Biology of *H. metabus*

The developmental cycle of *H. metabus* has three months duration, and thus four generations are reported each year. Each generation is almost synchronized and not overlapping with others, and the four generations appear regularly at fixed period during the year. In French Guiana, the adult moth emergences occur in January, April, July and October. The discrepancy of this cycle is exceptional, but may happen during periods with small densities. Nevertheless, during infestation periods (high densities), the presence of *H. metabus* females can persist for about four consecutive weeks (Vassal, 1989).

**Fig. 1.**
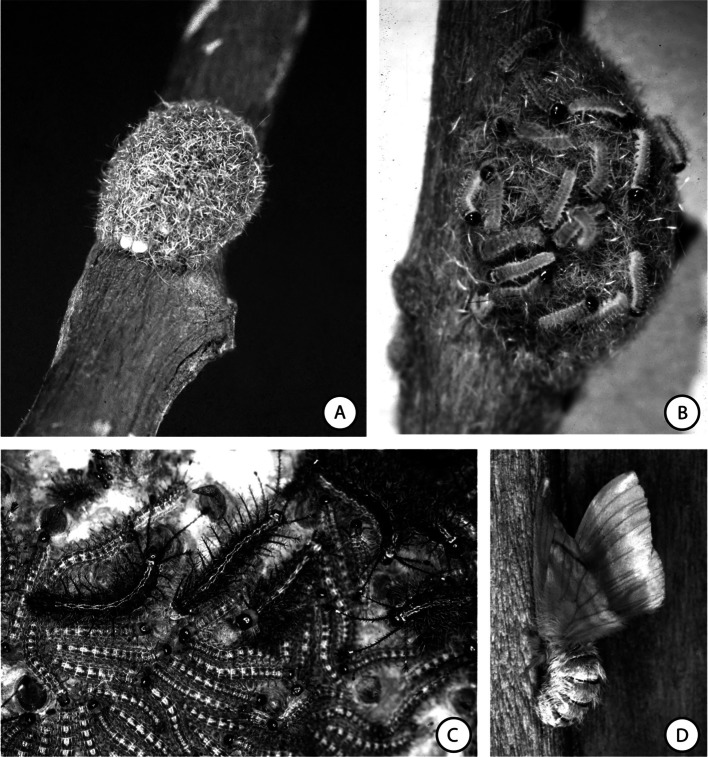
A. Egg-nest of *Hylesia metabus* covered by setae; B. Neewly hatched larvae of *Hylesia metabus*; C. Fourth and fifth instar larvae of *Hylesia metabus*; D. Adult female of *Hylesia metabus* adopting a defensive posture.

About 200 to 300 *H. metabus* eggs are deposited by a female in a single batch (Vassal, 1989). Immediately after the oviposition, the female covers the eggs with a layer of micro-arrow setae (Fig. 1). After 19 to 25 days, the eggs hatch and the larval development, that includes seven stages, has a duration varying between 40 to 50 days. During all larval stages the caterpillars are phytophagous, and gregarious allowing synchronized metamorphosis from one stage to the other until stage 4, for all individuals belonging to the same batch of eggs. During the first three months of its life, the caterpillar remains at the lower side of the leaves and the first larval stages feed only this lower-side leaving a transparent thin layer on the upper-side (Tisseuil, 1935). After the fourth larval stage, the feeding period (between 4 p.m. and 10 a.m.) takes turn with resting period on treetrunks during the warmer hours of the day (between 10 a.m. and 4 p.m). A processionary behavior has also been reported when the caterpillars leave the treetrunks, and patches of hundred or thousands of sameage caterpillars resting on shaded trunks were observed.

Between the larval and adult stages, the pupal stage has a duration of 15 to 20 days. The emergence of males occurs about 5 days before the female emergence. The adults have a short life of three to six days. As with all Saturniidae, the adult mouthparts are vestigial and digestive tracts are absent. Both sexes subsist without ingestion of food. During the daytime, the adult moths rest at ground level. The flying activity is crepuscular and just after sunset (7 to 8 p.m.). However, during strong infestations, the moths can be active until 11 p.m. The females are attracted by light, in particular the gravid females. White light is more attractive than yellow or orange lights. The adults can fly for several kilometres because caterpillars were found in the Devils’ Islands, situated at 11 km from the French Guiana coast (Vassal, 1985) and *H. metabus* populations went across the Gulf of Paria for 14 km, between Trinidad and Venezuela (Polar *et al.*, 2010). The eventual role of winds in such flights is unknown.

The mating of *H. metabus* is under the control of sex pheromones produced by females and attracting males. Electroantennographic studies have shown the biological activity on males of extracts from female abdominal apex (Herrera *et al.*, 2007). The pheromone composition has been investigated and some compounds were isolated and identified: 5-eicoseno, 1-octadecanol and 1-eicosanol (Liendo-Barandiaran *et al.*, 2007). In Venezuela, differences in the pheromones composition were found for populations from the State of Sucre and the State of the Delta Amacuro. These phenotypic differences suggest the existence of genetic differences. Mating takes place the first night after the female emergence, and the females are inseminated only once (Vassal, 1989). Other reproductive features such as mating behaviour, mating period, oviposition behaviour and primary sex-ratio were studied by Fornés and Hernández (2000).

### Ecology of *H. metabus*

In French Guiana, *H. metabus* is found both in the coastal zones of mangrove and in the inland forested areas. But, both adult populations present colour differences and relationships/exchanges between these populations are unknown. The massive infestations are only due to moths originating from mangrove areas. However, the forest population densities vary in the same way as the coastal populations even if they remain smaller (Vassal, 1989).

**Fig. 2.**
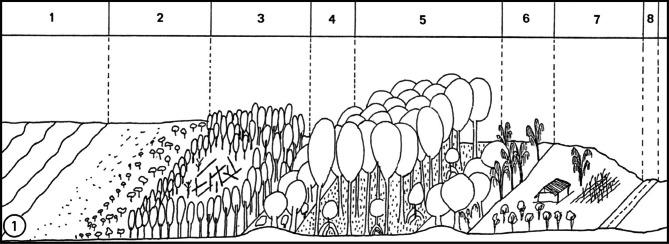
Graphical representation of the mangrove of Guatemala in French Guiana (Vassal, 1989). 1: Ocean; 2: Mud bank in sedimentation; 3: Young mangrove; 4: Sand zone from an old barrier beach; 5: Aged mangrove breeding site of *Hylesia metabus*; 6: Old barrier beach; 7: Cultivated area; 8: Road.
- *Rhizophora mangle*, *Rhizophora racemosa* and *Rhizophora harrisonii*, three species of the Rhizophoracea family, or red mangroves, characterized by their stilt roots and the red colour of their wood and sap. In French Guiana, the species of *Rhizophora* cannot be differentiated from morphological criteria out of the flowering season. These species are not adapted to salinity and are essentially found along the rivers, where they can exist away from the coasts. They develop tough leaves, rich in tannins, wax and secondary metabolites (Hernes *et al.*, 2001).

The mangroves are aquatic and forested ecosystems. In the inter-tropical Atlantic coastal zones, these ecosystems include at least ten species of tree-like plants and five of them are present in French Guiana (Guiral, 2002). These ecosystems are found in almost all the alluvial coastal zone (Fromard *et al*., 1988) representing an area of 140,000 hectares (about 1 % of the forest of French Guiana). The five mangrove species are:

- *Laguncularia racemosa* (Combretacea), mangrove tree of the seafront, colonizing the banks of mud originated from sedimentation and stabilization of the Amazonian mud and circulating along the French Guiana coast;
- *Avicennia germinans* (Avicenniacea) or white mangrove, the most abundant species of the French Guiana mangroves colonizing the more stable part of the mangrove, less submitted to the tides and swell influence. These white mangrove trees can reach 20 meters high and are characterized by a fast-growing potential and a stronger halotolerance. Consequently, *A. germinans* surpasses rapidly *L. racemosa* restricted to the smaller coastal fringe (Fig. 2). In French Guiana, the leaves biomass of *A. germinans*, with weak tannin concentrations, are the main feeding resource of *H. metabus* caterpillars;

The mangrove ecosystems of French Guiana have not been deeply investigated for their animal community either permanent or temporary. The aquatic fauna that uses the mangrove for feeding or protection was better described (Artigas *et al.*, 2003). The terrestrial fauna of mammals and birds, some of them more or less restricted to mangrove, were studied in a few areas. But, the invertebrate fauna and the insect fauna in particular remain mostly unstudied, with the exception of some blood-sucking Diptera such as Culicidae and Tabanidae and some social insects such as ants and termites that were studied for their implication in the litter mineralization process essential for the functional integrity of the mangrove ecosystem (Alongi, 1998; Guiral, 1999; Kathiresan & Bingham, 2001).

### Feeding preferences of *H. metabus*

During the periods where the *H. metabus* densities are small, the caterpillars feed almost exclusively on *A. germinans*. During the infestations periods and probably due to the lack of *A. germinans* material, the caterpillars become polyphagous (Silvain & Vassal, 1988): the caterpillars feed during several generations on other mangrove plant species such as *L. racemosa*, or even on savannah trees such as *Tapirira guianensis*, fruit trees such as *Citrus* sp and *Psidium guajava*. Vassal (1989) proposed a list of more than 25 plant species belonging to 16 families as feeding sources for *H. metabus* caterpillars during the infestations periods. Some of these plants are also found close to the human habitations.

In Venezuela *H. metabus* was reported to feed preferentially on the species *R. mangle* (Rodriguez-Acosta 1998; Fornés & Hernández, 2000; Hernández *et al.*, 2009), but in French Guiana the moth caterpillars have never being found on this red mangrove characterized by high tannins concentrations in leaves, much more than in *A. germinans* and *L. racemosa*. However, Tisseuil (1935) reported that caterpillars were accepting to feed on the red mangrove leaves in laboratory conditions.

### Infestations of *H. metabus*

The infestations are basically characterised by two processes. The emergence of adult moths is in densities much higher than usual. And, during the infestation periods, the moth dispersion is much extended than usual.

Infestations occur at irregular time intervals. The history of the infestations periods in French Guiana was reported by Vassal (1989) and four periods separated by about 20 years were identified: 1912-1918, 1931-1934, 1950, 1968. From the 70s and during the following 20years, the reporting of the infestation periods was more exhaustive and the infestations seemed more frequent after the 70s (Vassal, 1989). Then, between the infestation of 1989 (Vassal, 1989) and the report of 2006, the data were more fragmented. Recently, infestation episodes were reported at the beginning of 2006 (Renner & Girod, 2007) and in October 2007 (Service Santé Environnement de la DSDS de Guyane, 2008). Finally, the last infestation occurred between June and August 2011.

In Venezuela, the history of *H. metabus* infestations periods reported by Fornés and Hernández (2001) cannot be compared with the situation in French Guiana because observations were made for different periods, with the exception of the years 1997 to 1999, in which the exceptional infestations in Venezuela did not occur in French Guiana.

Our knowledge of the infestations of *H. metabus* in French Guiana is incomplete, in particular with the lack of epidemiological and entomological data. Globally, each infestation has about four weeks duration and includes two to three successive generations (Vassal, 1989).

Furthermore, the changes in the human society, in particular the economic development leading to different professional habits, have an impact on the human exposure to this Lepidopterism. Indeed, most of dermatitis cases are linked to anthropization or anthropic activity taking place in the close surrounding of the mangrove: port activities (Fornés & Hernández, 2001; Hassing & Bauer, 2008), airport (Germanetto, 1983), European Space Center of Kourou (Germanetto, 1983; Vassal, 1985), offshore oil facilities (Polar *et al.*, 2010) or oil tankers sailing along the coasts (Dinehart *et al.*, 1985; Hassing & Bauer, 2008).

The land settlement and the evolution of the French Guiana society are thus increasing the exposure of human populations to the *H. metabus* infestations, and this nuisance is now becoming a public health problem. In this last sense, the dermatitis due to *H. metabus* can be considered as an emerging disease with determinants found in the modification of the exposure context, mostly from anthropic and sociological origin and probably less from biological or ecosystemic changes. Furthermore, the human population acceptance of this nuisance is evolving. But, this phenomenon cannot be estimated due to the lack of sociological data on the nuisance perception, attitude and knowledge. These data should be collected among the different human communities of French Guiana.

### Natural factors regulating *H. metabus* populations

Predators of *H. metabus* caterpillars and pupae have been identified (Silvain & Vassal, 1991; Hernández *et al.*, 2009), among which the Reduviidae *Arilus cristatus* (Hemiptera: Reduviidae) was used as a biological control tool against the caterpillars in the Venezuelan mangrove (Osborn *et al.*, 2002). This Reduviidae species is also present in French Guiana but no predation on caterpillars was observed (Vassal, 1989). Another Reduviidae species, *Harpactor angulosus* was observed predating caterpillars of *H. metabus* in Brazil (Pereira *et al.*, 2009). Again, this Reduviidae species is also present in French Guiana.

In the Northeast of Venezuela, several parasitoids have also been identified on *H. metabus* such as the Diptera species *Belvosia* spp. (Tachinidae) and *Sarcodexia lambens* (Sarcophagidae) (Hernandez *et al*., 2009). They could be of some interest in the development of biological control tools. In French Guiana, Chalcidoidea (Hymenoptera) insects were observed emerging from the *H. metabus* eggs collected in the mangrove of Sinnamary in September 2011 and brought to the laboratory; the specimens were females only, belonging to the Eulophidae family (B. Pintureau, pers. com.).

Osborn *et al.* (2002) identified several bacteria pathogenic for *H. metabus* and suggested that these bacteria, in particular the best candidate *Pseudomonas aeruginosa*, should be vectorized through predators for a better use as a biological control tool.

The fungus *Beauveria bassiana* was also reported as pathogenic against *H. metabus*.

In French Guiana, Vassal (1989) identified virus (baculovirus) and bacteria from dead caterpillars collected in the field and brought to the laboratory. The bacteria was lately identified as *Bacillus thuringiensis israelensis* serotype H14 (Bti). Both virus and Bti could control the *H. metabus* populations, because during an infestation a 100 % mortality was observed on wild and laboratory bred caterpillars. A great proportion of the dead individuals were harbouring simultaneously the virus and the bacteria (Silvain & Vassal, 1991). This Bti strain isolated from field caterpillars was then tested under laboratory conditions for its pathogenic effect on *H. metabus*. The results have shown that *H. metabus* was very susceptible to this Bti (Vassal *et al.*, 1993). It was an unusual result for a Lepidoptera species since Lepidoptera are reputed to be not susceptible to *Bacillus thuringiensis israelensis* toxins. This finding may have important consequences on the development of new tools for the control of *H. metabus* and possibly other Lepidoptera.

Predation and parasitism do not appear as essential factors of regulation for the *H. metabus* populations. At the opposite, intrinsic epizootics were observed at the same time in mangrove and savannah breeding sites (Vassal, 1989). The insect diseases could be supported by the decrease of the feeding resources and could stop an infestation. The periodicity of the infestations, that may occur each six months to four years, remains of undetermined causes.

Finally, the mechanisms regulating the population dynamics of *H. metabus*, either when increasing or decreasing are largely unknown. No regulating abiotic factor such as hydro-sedimentation dynamics or climatic data was clearly identified, but meso-climatic factors could influence the *H. metabus* infestations that seem more important during the dry season.

## Control of *H. metabus*

### Light-traps

Nowadays, the light-traps are the best tools to limit the impact of the moth infestations on human populations. These traps are based on the attractivity of the light for the moths and for the gravid females in particular (Vassal, 1989). The light-traps can be placed such as a barrier between the mangrove where the moths are emerging and the human habitations. These traps are constructed from the classical moth-trap sampling model coupled with a killing device such as a water recipient containing detergent or waste oil where the moths drown, or a white cloth soaked with insecticide (deltamethrin) on which the moths are intoxicated.

During the light-trap use, other light sources (public and private lights) must be out or reduced to avoid the competition with the traps. For the best efficacy of the light-traps, a complete light extinction is necessary in the inhabited zones. The importance of these complementary measures has been emphasized by several authors (Silvain & Vassal, 1991; Iserhard *et al.*, 2007). The light-traps are the adult control tool the most efficient compare to the spraying of malathion or water with fire hose nozzle on walls where moths are resting (Vassal, 1985). However, the light power must be sufficient and the light emission must be included in wavelength between 500 to 550 nm, corresponding to a blue-green light, which is the most attractive for the moths (Franck, 1988). Indeed the moths are more attracted by mercury-vapour and metal halide lamps than sodium-vapour lamps, even if high-pressure sodium lamps have a large spectrum and emit in the green as well. The low-pressure sodium lamps, in the contrary have a narrow spectrum, centred on the 598 nm wavelength and emit on orange, being thus less attractive for the moths. So, when it is not possible to extinct all light sources during the periods of females activity, it is recommended to use light sources of low intensity with emission on orange. Finally, if the number of light-traps has to be sufficiently important to attract as much as possible *H. metabus* females, these traps must also be placed at reasonable distances from each other to avoid competition and lost of confused moths wandering between traps. The same moth confusion was observed when moonlight decreases light-trap attractivity (Robert, 1980).

### Control of the adults with insecticide

Deltamethrin spraying is usually forbidden in humid zones due to its ecological impact and toxicity for aquatic fauna (shellfish and fish in particular). But, this insecticide can be used in areas distant of the humid zones and could be added to the control tools, at least in special cases such as the control of the adults resting during the day on the buildings’ walls (either outdoors or indoors), or the control of the moths wandering in the vicinity of the light-traps (Vassal, 1989; Glasser *et al.*, 1993). This insecticide is also used to soak the cloth of some light-traps (see above).

Other insecticides could also be used such as pyrethroids for their knock down effect. But substances or formulations enhancing the micro-arrow setae dispersion through exciting behaviour response of the moths, such as domestic sprays, are not recommended.

### Control of the larval stages

In the past, extended pyrethroid sprayings were carried out in the French Guiana mangrove with negative ecological consequences, in particular for the aquatic fauna (Vassal, 1989). Actually, the large scale treatment in field conditions are authorized only with very selective products. The insecticides of microbiological origin (such as *Bacillus thuringiensis*) are now the only ones matching these criteria. Whatever the product, the more susceptible younger stages should be the target of the treatment. And, as a consequence, all control action must be based on an entomological surveillance system. The microbiological products actually available are:

- *Bacillus thuringiensis kurstaki* serotype H3a3b (Btk), known to be active against Lepidoptera and widely used in plant protection against the phytophagous caterpillars found on vegetables, fruits, ornamental plants and forests. Btk is presently used against the pine and oak processionary caterpillars as well as other defoliators Lepidoptera (van Frankenhuyzen, 1990; Bauce *et al.*, 2004; Cebeci *et al.*, 2010). Btk is also used in Argentina against another urticant Lepidoptera of the genus *Hylesia*, *H. nigricans* (Salomon *et al*., 2005). In French Guiana, Btk was sprayed several times for the control of *H. metabus* caterpillars with aerial, helicopter and microlighting sprayings and some operational recommendations could be estimated for formulation, dose, period and efficacy (Vassal, 1989). This type of control is also efficiently implemented in Venezuela with information on the cost of the treatment (Osborn, 2002). Other results on the formulation, efficacy of Btk and most susceptible larval stages were succinctly reported (Osborn *et al.*, 2005b). But, other data showed the negative impact of Btk on the fauna, in particular on the Lepidoptera family of Sphingidae (Clavijo *et al.*, 2007) and Nymphalidae (Arias *et al.*, 2007). It seems worth mentioning herein the regulatory framework of the use of Btk in Europe and therefore in French Guiana. On one hand, products containing Btk can be used as a plant protection product. The control of oak processionary caterpillars (*Thaumetopoea processionea*) falls into this category and is therefore intended – from a regulatory point of view – to protect forestry. On the other hand, biocidal products containing Btk as active substance are currently not authorized in the European Union for an insecticide use in sanitary purposes (Product-Type n° 18 according to the European legislation on biocidal products). Therefore, the Btk can not currently be used for the control of *H. metabus* in the mangroves of French Guiana.
- *Bacillus thuringiensis israelensis* serotype H14, (Bti) strain isolated from dead caterpillars of *H. metabus* (Vassal, 1989). The pathogenicity of Bti toxins is well known for larvae of some species of Diptera such as the mosquitoes, but these toxins are generally nonpathogenic for Lepidoptera caterpillars. The pathogenic effect of Bti on *H. metabus* was surprising but demonstrated under laboratory conditions (Vassal *et al.*, 1993). Furthermore, the same studies have shown that the Bti (H14) strain isolated from field caterpillars was more pathogenic compared to the commercial strain of Btk (H3a3b). However, before any field treatment with Bti, further studies are needed to complete the available data on commercial Bti (Vassal, 1993). The surprising efficacy of Bti against *H. metabus* deserves further laboratory and field investigations, to precise the working concentrations and the susceptible larval stages. The pathogenic effect of the product could also be increased through a formulation allowing the insecticide fixation on the leaves of the trees where caterpillars are feeding and resting (this type of formulation is actually not available).

## Research for an imrovement of the surveillance and control of *H. metabus* infestations

### Development of a targeted and efficient control

Apart from the use of light-traps, the control of larvae represents the best option to prevent the *H. metabus* infestations, and Btk is actually the product of reference for this type of action. For an optimal efficacy, the younger larval stages should be targeted. However, the use of Bti could represent a very interesting opportunity, leaving safe the other Lepidoptera. The evaluation under natural conditions of Bti efficacy against the *H. metabus* caterpillars is thus a priority. But, its use is not easy due to the absence of commercial formulation adapted to caterpillars on tree leaves and precise definitions of the operational methods. Whatever the product, Btk or Bti, its environmental impact can be estimated only with a best knowledge of the mangrove fauna. It is also essential to note that all control action must respect the French and European regulations and this obligation can determine the choice of control methods regarding products and formulation authorizations.

Within the surveillance systems, the male feature of emergence a few days before females could be used through a pheromone-trap system, because *H. metabus* females emit a sexual pheromone that could be synthesized. This device may provide an interesting warning tool.

At the opposite, it is difficult to imagine a pheromonetrap that could create enough sexual confusion in males or collect enough males to negatively influence the density of the following generation, due to the high population densities observed during the infestation periods (Shea, 1995).

In a long-term overview, it appears fundamental to study the determinism of the *H. metabus* infestations, including the genetics, to improve the global control strategy. These studies must also include the dynamics of the ecosystems colonized by the caterpillars as well as the functional relationships between *H. metabus* populations and the mangrove system. Evolutionary questions (genetic flows, speciation, etc.) are of great interest considering the stable forest populations of *H. metabus*. Remote sensing modelling could also provide better spatial approach of the phenomenon and infestations simulations, with a special interest for areas where access is difficult.

### Social determinants of *H. metabus* control

The social approach is an important element of the control strategies. Recent analyses have shown that the nuisance/vector prevention/control efficacy is widely based on the human population perception and acceptability of the actions implemented by public authorities. The exposure of humans to the *H. metabus* infestations is related not only to the moth densities but also to anthropic determinants such as collective behaviours and ways of life. These last factors are evolving with economical development, land settlement, social progress and the natural expectation of a better life level. Information campaigns and heath education promote the development of preventive attitudes that can reduce the contact between the urticant moth setae and human populations: reduction of light sources, regular cleaning of furniture exposed to the moth setae, wearing of protecting clothes (Rodriguez-Morales *et al*., 2005; Polar *et al.*, 2010).

In another hand, the social demand for environment preservation can also be antagonist with the population request of nuisance control. Consequently, the implementation of long-lasting measures, accepted by the human populations, necessitates to take into account several factors, cognitive, psychological and sociological, and to initiate a participative process for the conception of control management. Garcia *et al.* (2009) carried out studies on the knowledge and attitudes of the human populations related to *H. metabus* infestations, in the State of Amacuro delta in Venezuela. Apart information on the local perception, the study strongly suggested the community participation in the surveillance system of the mangrove colonized by the caterpillars and also of the domestic plants.

In French Guiana, no information on the human population perception of the health risk due to *H. metabus*, or even on the moth control, is available. The implementation of a control strategy accepted by local populations necessitates studies on the local perceptions and attitudes for this nuisance, as well as for the available control methods. Taking into account this social dimension may also promote preventive practices both collective and personal.

### Implementation of a surveillance and warning system

In French Guiana, an entomological warning system allowing the early implementation of *H. metabus* measures is a necessity. This alert will be helpful not only for control actions, but also for surveillance of epidemiological data. The results will then be used in the estimation of the public health burden due to *H. metabus* infestations and in the evaluation of the control actions efficacy. The entomological surveillance may be based on the observation of caterpillar patches and/or adult traps in mangrove swamps and around human settlements. A surveillance system proposed by Renner & Girod (2007) is considered as a first approach, easy and simple, that could be used immediately to survey the *H. metabus* infestations. This approach will be improved through field studies and best-adapted trapping-methods may be identified. Critical threshold are also needed to decide outbreaks and control actions.

Periodical flight above the mangrove breeding sites may be helpful in determining through infrared captors the leaves biomass indicating the level of feeding of the caterpillars on the *Avicennia* species. This indicator could be related to the infestations importance and timing.

## Conclusive comments

In conclusion, it is stringent that the dermatitis called “papillonite” is at the intersection of three disciplines: (i) medical entomology for the public health impact of the dermatitis, (ii) agricultural and forestry entomology for the feeding of the tree leaves by the caterpillars, in particular the white mangrove trees, and (iii) the political ecology (Robbins, 2004). This last field refers to the fact that complete protection of human populations against this nuisance/ health problem due to *H. metabus* is antagonist with the absolute protection of the environment including patrimonial areas such as the mangroves.

Choices have to be made, and if both protection strategies were placed distantly in a straight line, it is to the human populations to position the cursor between both strategies, taking into account all the aspects of the problem including the financial ones. The number of public stakeholders at different level of the sociopolitic structure of French Guiana (State, Region, Department, National Park, inhabitants, tourists, vector control agency, fishermen, hunters, nature protection agencies, etc.) will make difficult a consensus that is obviously evolutional.

This assessment emphasizes the necessity of interdisciplinary scientific collaboration in the preliminary studies and recommendations.

## References

[R1] Alongi D.M. Mangroves and salt marshes. *in*: Coastal ecosystem processes, CRC Press, Boca Raton, FL, USA, 1998, 43–92

[R2] Arias Q., Clavijo J.A., Herrera M., Osborn F. & Desousa J.C. Efecto de aplicaciones de *Bacillus thuringiensis* var. *kurstaki*, sobre la diversidad de especies de Nymphalidae (Insecta: Lepidoptera) en el Golfo de Paria estado Sucre, Venezuela, Resumen n°51. XX Congreso Venezolano de Entomología. Entomotropica, 2007, 22, 74

[R3] Artigas L.F., Vendeville P., Leopold M., Guiral D., Ternon J.F. Marine biodiversity in French Guiana: estuarine, coastal, and shelf ecosystems under the influence of Amazonian waters. Gayana, 2003, 67, 302–326

[R4] Battisti A., Holm G., Fagrell B. & Larsson S. Urticating hairs in arthropods: their nature and medical significance. Annual Review of Entomology, 2011, 56, 203–220 10.1146/annurev-ento-120709-144844 20809805

[R5] Bauce E., Carisey N., Dupont A. & van Frankenhuyzen K. *Bacillus thuringiensis* subsp. *kurstaki* aerial spray prescriptions for balsam fir stand protection against spruce budworm (Lepidoptera: Tortricidae). Journal of Economic Entomology, 2004, 97, 1624–1634 15568352 10.1603/0022-0493-97.5.1624

[R6] Benaim-Pinto C. Reacciones cutaneas indeseables producidas por insectos. Dermatologia venezolana, 2002, 40, 87–94

[R7] Boyé R. La papillonite guyanaise. Bulletin de la Société de Pathologie Exotique, 1932, 25, 1099–1107

[R8] Cebeci H.H., Oymen R.T. & Acer S. Control of pine processionary moth, *Thaumetopoea pityocampa* with *Bacillus thuringiensis* in Antalya. Turkey. Journal of Environmental Biology, 2010, 31, 357–361 21047011

[R9] Centre National D’Expertise sur les Vecteurs Réponse à la saisine “Stratégies et méthodes de lutte optimales contre *Hylesia metabus*”, agent de la papillonite en Guyane française. Avis à l’attention de la Direction Générale de la Santé CNEV, Montpellier, 12 août, 2011 21 p + annexe

[R10] Clavijo J.A., Chacín M.E., Arias Q., Osborn F. & Herrera M. Efecto de aplicaciones de *Bacillus thuringiensis* var. *kurstaki*, sobre la diversidad de especies de Sphingidae (Insecta: Lepidoptera) en el Golfo de Paria, estado Sucre, Venezuela, Resumen n°49. XX Congreso Venezolano de Entomología. Entomotropica, 2007, 22, 73–74

[R11] Diaz J.H. The evolving global epidemiology, syndromic classification, management, and prevention of caterpillar envenoming. American Journal of Tropical Medicine and Hygiene, 2005, 72, 347–357 15772333

[R12] Dinehart S.M., Jorizzo J.L. & Soter N.A. Evidence for histamine in the urticating hairs of *Hylesia* moths. The Journal of Investigative Dermatology, 1987, 88, 691–693 3585053 10.1111/1523-1747.ep12470352

[R13] Dinehart S.M., Archer M.E., Wolf J.E., McGavran M.H. Reitz C. & Smith E.B. Caripito itch: dermatitis from contact with *Hylesia* moths. Journal of American Academy of Dermatology, 1985, 13, 743–747 10.1016/s0190-9622(85)70216-2 4078069

[R14] Ducombs G., Lamy M., Pradinaud R., Jamet P., Voncendeau P., Maleville J. & Texier L. La papillonite de Guyane française. Étude clinique et épidémiologique. Annales de Dermatologie et de Vénéréologie, 1983, 110, 809–816 6666924

[R15] Fornés L. & Hernández J.V. Algunos aspectos de la biología de *Hylesia metabus* (Cramer 1775) (Lepidoptera: Saturniidae). Boletín de Entomología Venezolana, 2000, 15, 127–145

[R16] Fornés L. & Hernández J.V. Reseña histórica e incidencia en la salud pública de *Hylesia metabus* (Cramer) (Lepidoptera: Saturniidae) en Venezuela. Entomotropica, 2001, 16, 137–141

[R17] Franck K.D. Impact of outdoor lighting on moths: an assessment. Journal of the Lepidopterists’ Society, 1988, 42, 63–93

[R18] Fromard F., Puig H., Mougin E., Marty G., Betoulle J.L. & Cadamuro L. Structure, above-ground biomass and dynamics of mangrove ecosystems: new data from French Guiana. Oecologia, 1988, 115, 39–53 10.1007/s004420050489 28308466

[R19] Garcia Z.B., Alvarado P.G. & Lopez de Aguilar R. Conocimientos y prácticas sobre *Hylesia metabus* (Cramer, 1775) y lepidopterismo en Capure, estado Delta Amacuro, Venezuela (Julio-Agosto 2005). Boletín de Malariología y Salud Ambiental, 2009, 49, 293–301

[R20] Germanetto P. Guyanese moth allergy in French Guiana. Pan American Health Organization Bulletin, 1983, 17, 304–306

[R21] Glasser C.M., Cardoso J.L., Carréri-Bruno G.C., Domingos M.F., Moraes R.H.P. & Ciaravolo R.M.C. Surtos epidêmicos de dermatite causada por mariposas do gênero *Hylesia* (Lepidoptera: Hemileucidae) no Estado de São Paulo. Brasil. Revista de Saúde Pública, 1993, 27, 217–220 10.1590/s0034-89101993000300011 8115837

[R22] Guiral D. Les écosystèmes à mangrove, *in*: Rivières du Sud. Sociétés et mangroves ouest-africaines, Cormier-Salem M.C, IRD Éditions, Paris, 1999, 65–130

[R23] Guiral D. De vasière à mangrove et de mangrove à crevettes. *in*: Guyane ou le voyage écologique, Richard-Hansen C. & Le Guen R., Éditions Roger Le Guen, Garies, France, 2002, 196–201

[R24] Hassing R.J. & Bauer A.G.C. Pruritic dermatitis on an oil tanker after a visit to French Guyana. Journal of Travel Medicine, 2008, 15, 464–465 19090805 10.1111/j.1708-8305.2008.00260.x

[R25] Hernández J.V., Osborn F., Herrera B., Liendo-Barandiaran C., Perozo J. & Velásquez D. Parasitoides larva-pupa de *Hylesia metabus* Cramer (Lepidoptera: Saturniidae) en la región nororiental de Venezuela: un caso de control biológico natural. Neotropical Entomology, 2009, 38, 243–250 19488514 10.1590/s1519-566x2009000200012

[R26] Hernes P.J., Benner R., Cowie G.L., Goñi M.A., Bergamaschi B.A., Hedges J.I. Tannin diagenesis in mangrove leaves from a tropical estuary: a novel molecular approach. Geochimica et Cosmochimica Acta, 2001, 65, 3109–3122

[R27] Herrera B., Liendo-Barandiaran C.V. & Hernández J.V. Electroantenografía de extractos ápices abdominales de *Hylesia metabus* (Lepidoptera: Saturniidae), Resumen n°227. XX Congreso Venezolano de Entomología. Entomotropica, 2007, 22, 135

[R28] Hossler E.W. Caterpillars and moths: Part II. Dermatologic manifestations of encounters with Lepidoptera. Journal of the American Academy of Dermatology, 2010, 62, 13–28 20082887 10.1016/j.jaad.2009.08.061

[R29] Hudson J.E. Trials of yellow lamps for reducing plagues of itch moths in Suriname. Institution of Public Lighting Engineers Lighting Journal, 1985, 50, 42–44

[R30] Iserhard C.A., Kaminski L.A., Marchiori M.O., Teixeira E.C. & Romanowski H.P. Occurrence of Lepidopterism caused by the moth *Hylesia nigricans* (Berg) (Lepidoptera: Saturniidae) in Rio Grande do Sul State, Brazil. Neotropical Entomology, 2007, 36, 612–615 17934630 10.1590/s1519-566x2007000400022

[R31] Kathiresan K. & Bingham B.L. Biology of mangroves and mangrove ecosystems. Advances in Marine Biology, 2001, 40, 81–251

[R32] Lamy M., Michel M., Pradinaud R., Ducombs G. & Maleville J. L’appareil urticant des papillons *Hylesia urticans* Floch et Abonnenc, et *H. umbrata* Schauss (Lépidoptères : Saturniidae) responsables de la papillonite en Guyane française. International Journal of Insect Morphology and Embryology, 1982, 11, 129–135

[R33] Lamy M. & Lemaire C. Contribution à la systématique de *Hylesia* : étude au microscope électronique à balayage des “fléchettes urticantes”. Bulletin de la Société Entomologique de France, 1983, 88, 176–192

[R34] Lamy M., Pastureaud M.H., Novak F. & Ducombs G. Papillons urticants d’Afrique et d’Amérique du Sud (genus *Anaphae* et genus *Hylesia*): contribution du microscope électronique à balayage à l’étude de leur appareil urticant et à leur mode d’action. Bulletin de la Société Zoologique de France, 1984, 109, 163–177

[R35] Lamy M. & Werno J. The brown-tail moth of Bombyx *Euproctis chrysorrhoea* L. (Lepidoptera) responsible for lepidopterism in France: biological interpretation. Comptes rendus de l’Académie des sciences. Série III, Sciences de la vie, 1989, 309 (14), 605–610 2510913

[R36] Léger M. & Mouzels P. Dermatose prurigineuse déterminée par des papillons Saturnides du genre *Hylesia*. Bulletin de la Société de Pathologie Exotique, 1918, 11, 104–107

[R37] Lemaire C. The Saturniidae of America: Hemileucinae, Antiquariat Geock & Evers Germany, 2002, Part A, 688

[R38] Liendo-Barandiaran C.V., Herrera B. & Hernández J.V. Estudio de la feromona sexual de *Hylesia metabus* (Lepidoptera: Saturniidae), Resumen n°228. XX Congreso Venezolano de Entomología. Entomotropica, 2007, 22, 135

[R39] Lundberg U., Osborn F., Carvajal Z., Gil A., Guerrero B. & Piñango C.L.A. Isolation and partial characterization of a protease with kallikrein-like activity from the egg-nets of *Hylesia metabus* (Crammer 1775) (Lepidoptera: Saturniidae), Preliminary communication. Revista Cientifica, 2002, 12, 97–102

[R40] Lundberg U., Salazar V., Tovar M. & Rodriguez J. Isolation and partial characterization of proteins with vasodegenerative and proinflammatory properties from the egg-nests of *Hylesia metabus* (Lepidoptera: Saturniidae). Journal of Medical Entomology, 2007, 44, 440–449 17547229 10.1603/0022-2585(2007)44[440:iapcop]2.0.co;2

[R41] Novak F., Pelissou V. & Lamy M. Comparative morphological, anatomical and biochemical studies of the urticating apparatus and urticating hairs of some Lepidoptera: *Thaumetopoea pityocampa* Schiff., *Th. processionea* L. (Lepidoptera, Thaumetopoeidae) and *Hylesia metabus* Cramer (Lepidoptera, Saturniidae). Comparative Biochemistry and Physiology Part A: Physiology, 1987, 88, 141–146

[R42] Osborn F., Berlioz L., Vitelli-Flores J., Monsalve W., Dorta B. & Lemoine V.D. Pathogenic effects of bacteria isolated from larvae of *Hylesia metabus* Crammer (Lepidoptera: Saturniidae). Journal of Invertebrate Pathology, 2002, 80, 7–12 12234536 10.1016/s0022-2011(02)00037-x

[R43] Osborn F. La Palometa Peluda (*Hylesia metabus*) como problema. Creación del grupo multidisciplinario interinstitucional para su estudio y control, *in*: Sociedad Venezolana de Microbiología, Capítulo Sucre XXIX Jornadas Venezolanas de Microbiología “Dr. Vidal Rodríguez Lemoine”, Cumaná del 9 al 11 de Noviembre de 2005a, 11 p.

[R44] Osborn F., Hernández J.V., Velásquez D., Velásquez Y. & Sapene A. Efectividad de dos formulaciones del larvicida Dipel 8L® sobre larvas de *Hylesia metabus* Cramer (Lepidoptera: Saturniidae), Resumen n°158. XIX Congreso Venezolano de Entomología. Entomotropica, 2005b, 20, 176–177

[R45] Paniz-Mondolfi A.E., Pérez-Alvarez A.M., Lundberg U., Fornés L., Reyes-Jaimes O., Hernández-Pérez M. & Hossler E. Cutaneous lepidopterism: dermatitis from contact with moths of *Hylesia metabus* (Cramer 1775) (Lepidoptera: Saturniidae), the causative agent of caripito itch. International Journal of Dermatology, 2011, 50, 535–541 21506967 10.1111/j.1365-4632.2010.04683.x

[R46] Pelissou V. & Lamy M. Le papillon cendre : *Hylesia metabus* Cramer (= *H. urticans* Floch et Abonnenc) (Lepidopteres Saturnidae) papillon urticant de Guyane Française. Étude cytologique de son appareil urticant. Insect Science and its Application, 1988, 9, 185–189

[R47] Pereira A.I.A., Zanuncio J.C., Gill-Santana H.R., Ramalho F.S., Leite G.L.D. & Serrao J.E. *Harpactor angulosus* (Reduviidae: Harpactorinae), a predator of neotropical saturniids. *Hylesia* spp. in Brazil. Entomological news, 2009, 120, 206–212

[R48] Polar P., Cock M.J.W., Frederickson C., Hosein M. & Krauss U. Invasions of *Hylesia metabus* (Lepidoptera: Saturniidae, Hemileucinae) into Trinidad, West Indies. Living World, Journal of Trinidad and Tobago Field Naturalists’ Club, 2010, 1–10

[R49] Renner J. & Girod R. Propositions pour la surveillance entomologique de la papillonite en Guyane. Direction de la Santé et du Développement Social de Guyane, et Institut Pasteur de Guyane. Document N° 005/IPG/UEM/2007, 2007, 44 pp.

[R50] Robbins P. Political ecology: a critical introduction, Wiley- Blackwell, Oxford, 2004, 264 p.

[R51] Robert J.C. L’activité de vol nocturne de Lépidoptères appartenant à diverses familles. Annales Scientifiques de l’Université de Besançon, 4^*ème*^ sér., 1980, 1, 3–20

[R52] Rodriguez J., Hernández J.V., Fornés V., Lundberg U., Piñango C.L.A. & Osborn F. External morphology of abdominal setae from male and female *Hylesia metabus* adults (Lepidoptera: Saturniidae) and their function. Florida Entomologist, 2004, 87, 30–36

[R53] Rodriguez-Acosta A., Rubiano H., Reyes M. & Fernández S.T. *Dermatitis causada* por *Hylesia metabus* (Lepidoptera: Hemilucidae) en la región costera del Estado del Delta del Amacuro Venezuela. Revista Cubana de Medicina Tropical, 1998, 50, 215–217 10349447

[R54] Rodriguez-Morales A.J., Arria M., Rojas-Mirabal J., Borges E., Benitez J.A., Herrera M., Villalobos C., Maldonado A., Rubio N. & Franco-Paredes C. Lepidopterism due to exposure to the moth *Hylesia metabus* in Northeastern Venezuela. American Journal of Tropical Medicine and Hygiene, 2005, 73, 991–993 16282317

[R55] Salomón O.D., Simón D., Rimoldi J.C., Villaruel M., Pérez O., Pérez R. & Marchan H. Lepidopterismo por *Hylesia nigricans* (mariposa negra) investigacion y accion preventiva en Buenos Aires. Medicina (Buenos Aires), 2005, 65, 241–246 16042136

[R56] Service Santé Environnement de la DSDS de Guyane Bilan de la surveillance de la papillonite en Guyane, réalisée à partir du réseau des pharmaciens (octobre-novembre 2007). Bulletin d’Alerte et de Surveillance Antilles Guyane, 2008, 2, 6–7

[R57] Shea P.J. Use of insect pheromones to manage forest insects: present and future. Biorational Pest Control Agents, 1995, 595, 272–283

[R58] Silvain J.F. & Vassal J.M. Historique et perspectives des travaux réalisés par l’ORSTOM en Guyane sur certains facteurs biotiques de régulation des populations d’insectes et leur utilisation en lutte intégrée. Rencontres Caraïbes en lutte biologique, Guadeloupe, 5–7 novembre 1990 Les Colloques de l’INRA, 1991, (58), 513–527

[R59] Sociedad Venezolana de Entomología Resúmenes del XIX Congreso Venezolano de Entomología. Entomotropica, 2005, 20, 127–204

[R60] Sociedad Venezolana De Entomología Resúmenes del XX Congreso Venezolano de Entomología. Entomotropica, 2007, 22, 57–143

[R61] Thiéry G., Adam S., Coulet O., André N., Meynard J.B. & Thiéry S. Papillonite. Médecine Tropicale, 2008, 68, 27–28 18478767

[R62] Tisseuil J. Contribution à l’étude de la papillonite guyanaise. Bulletin de la Société de Pathologie Exotique, 1935, 28, 719–721

[R63] Van Frankenhuyzen K. Development and current status of *Bacillus thuringiensis* for control of defoliating forest insects. The Forestry Chronicle, 1990, 66 (5), 498–507

[R64] Vassal J.M. Programme d’étude de la biologie et de l’écologie de l’agent de la papillonite en Guyane Française, en vue de la mise en place d’une structure de lutte intégrée. Centre ORSTOM de Cayenne, Novembre, 1985, 32 p.

[R65] Vassal J.M. Biologie, écologie et pathologie d’*Hylesia metabus* (Cramer 1775) (Lépidoptères : Saturniidae) agent de la “papillonite” en Guyane Française : mise en place d’une structure de lutte intégrée [Thèse de Sciences]. Université des sciences et techniques du Languedoc Montpellier, 1989, 248 p. + 44 pl

[R66] Vassal J.M., Dauthuille D. & Silvain J.F. *Hylesia metabus*, agent de la papillonite en Guyane Française. *In*: Le littoral Guyanais. Congrès Sepanguy-Sepanrit, Cayenne, 1986, 125–130

[R67] Vassal J.M., de Barjac H., Frutos R. & Federici B.A. Isolation of *Bacillus thuringiensis* subsp. *israelensis* from diseased field-collected larvae of the saturniid moth, *Hylesia metabus*, in French Guiana. Federation of European Microbiological Societies Microbiology Letters, 1993, 107, 199–204

[R68] Willat G., Capdevila A., Martinez M., Benavides C. & Carballo R. Brotes de dermatitis urticante por mariposas del género *Hylesia* em Uruguay. Revista Salud Pública (Bogotá), 2003, 2, 4–6

